# Anticancer Activity of Solvent Extracts of *Hexogonia glabra* against Cervical Cancer Cell Lines

**DOI:** 10.31557/APJCP.2020.21.7.1977

**Published:** 2020-07

**Authors:** Swapan Kumar Ghosh, Tapojyoti Sanyal, Tanmay Bera

**Affiliations:** *Molecular Mycopathology Lab, Cancer Research Unit, PG Department of Botany, Ramakrishna Mission Vivekananda Centenary College (Autonomous), Rahara, Kolkata 700118, India. *

**Keywords:** Human cancer cell line, mushroom, apoptosis, cytotoxicity, MTT assay

## Abstract

**Objective:**

In this study, we aimed to harness some solvent extracts of one wild mushroom *Hexagonia glabra* and test their anti-cancer activity against cervical human cell lines, namelyHeLa, SiHa, and CaSki.

**Methods:**

It includes cell morphological study by microscope, nuclear morphology by DAPI staining under fluorescence microscopy, apoptosis assay by fluorescence technique, anti-proliferation by MTT assay and expression of apoptotic and anti-apoptotic genes by Western blotting and cell cycle analysis was done.

**Results:**

The selected cervical cancer cells were treated separately with 150 µg/mL of three extracts, namely of ethanolic (EE), ethyl acetate (EAE), and water extract (WE) and exhibited features like round, shrink and dead. All extracts caused apoptosis in cell lines and EE had the highest effect in this regard. The percentages of apoptotic cells in HeLa, SiHa and CaSki, at the same concentration of EE were 79.23, 75.42, and 76.36% respectively. Cytotoxicity assay showed that all three extracts (50 – 250 μg/mL) were potent for inhibition of cell growth of three cell lines and again EE had the highest effect. The percentages of cell growth inhibition in HeLa, SiHa, and CaSki cells treated with EE at 24 h at 50 µg/mL were 45.79±4.11, 41.66±4.03, and 36.72±2.67, while they were 74.23±7.45, 62.31±5.97, and 54.23±5.04 at 150 µg/mL concentration. At 250 µg/mL concentration, the percentages of cell growth inhibition were 94.25 ±8.11, 90.02 ±8.67, and 85.43±6.21, respectively. The expression of apoptotic gene (*Caspase 3, 9*) and tumor guard gene (*p53*), as their proteins in Western blotting increased . However, anti-apoptotic *BcL2* gene of all cell lines was decreased following treatment with extracts. In addition, the cell cycle analysis (CaSki cell) showed that treatment (EE) arrested at G2/M check point cell cycle.

**Conclusion:**

All extracts of this mushroom were active in arresting growth of three cell lines and EE had the highest effect, indicating that this mushroom can be a valuable source of anticancer agents.

## Introduction

Cancer is now one of the most complex killer diseases among human beings. Hurdles and hopes for cancer treatment are going on, but scientists search the new natural compound that can be applied as an anticancer remedy (Hunt, 2002). Searching for natural sources to find novel bioactive anticancer compounds may provide next generation of drugs (Liu et al., 2010; Lindequist et al., 2010; Xu et al., 2011; Aly et al., 2011). Mushrooms are macro-fungi whose so-called fruit bodies are seen under naked eyes. Nowadays, scientists have paid much attention to mushrooms in cancer management because they have hundreds of novel natural constituents with biological properties with lower toxic side effects and even lower cost (Lee et al., 2014; Shavit et al., 2009). In addition, mushroom extracts can act as immunomodulatory factors in the management of cancer patients (Ghosh and Chakrabarty, 2018). Lucas et al., (1957) for the first time showed that mushrooms had anticancer property. After intensive research, some anticancer drugs, Lentinan from fruiting bodies of *Lentinus edodus*, Krestin from cultured mycelium of *Trametes versicolor*, and Schizophyllan from fruit body of *Schizophyllum commune*, were introduced for cancer management (Mizuno, 1999; Wasser, 2002; Mo, 2004). Some mushroom products like Grifolan from *Grifola frondosa* exhibit anti cancer activity against breast, gastrointestinal, liver, and lung cancers (Poucheret et al., 2006) by activating macrophage which triggers cytokine secretion. SSG , a homoglucan from *Sclerotinia sclerotiorum*, triggers the development of TH1 cells via the IL-12 pathway. It is also isolated from *Sparassis crispa* and usually enhances the hematopoietic response (Moradali et al., 2007). Some β-glucans from medicinal mushrooms are responsible to activate both cell-mediated and humoral immunity by triggering different immune cells (T –cells, B- cells, macrophage, etc), hence rejecting tumor cells (Ladanyi et al., 1993; Kim et al., 1996; Kurashige et al., 1997; Brown and Gordon, 2003). The activated macrophages also secrete cytokines that prime natural killer (NK) cells and T lymphocytes, both of which are cytotoxic to tumor cells (Prestwich et al., 2008). There are more than 14,000 mushrooms out of 5.1 million estimated fungi (Blackwell, 2011), among which near about 700 exhibit medicinal properties (Wasser, 2011). In India, described mushroom species are 850 (Deshmukh, 2004; Manoharachary et al., 2005). Although the rate of application of fungi in different sectors has been increased exponentially throughout the world, near about 90% of fungal species are not yet screened in medical application like anticancer drug development. Among edible mushrooms, members of* Agaricus* are produced maximum, whilst from the non-edible medicinal mushroom *Ganoderma* are produced and used maximum globally. Many members of the Polyporaceae family have been now selected as the next candidate producers of possible valuable medicines (Mizuno, 1995). In India particularly in West Bengal, mushrooms are abundant but systematical screening of those as anti tumor or cancer has not been investigated by any one yet. On the other hand, every year in India, 122,844 women are diagnosed with cervical cancer, and 67,477 die from this disease (ICO, 2014; Sreedevi et al., 2015). The present treatments for cancer include chemotherapy, surgery, and radiation. All of these treatment approaches are associated with some complications. For instance, chemotherapeutic drugs are becoming narrow potential; on the other hand, patients are becoming resistant to these drugs (Ghosh, 2018). Under such situation, it is highly essential to search for new natural anticancer compounds, which are target specific and immune- enhancer. The screening of different mushroom extracts and isolation of different natural compounds and their targeted anticancer effects with immune enhancing property are need of hour. In this study, we tested wild *Hexogonia glabra* mushroom extracts, such as ethanolic (EE), ethyl acetate (EAE), and water extract (WE), as anticancer agents against cervical cell lines (HeLa, SiHa and CaSki).

## Materials and Methods

*Mushroom collection*

Fruiting bodies of wild mushroom, *Hexagonia glabra *(P. Beauv.) Ryvarden (Family: Polyporaceae), were collected in September 2018 from different wooden logs from various zones of south Twenty Four Parganas district, India. The samples were sent to laboratory, and their identification was confirmed by consulting with the published keys (Ryvarden, 1999; Watling, 1973; Pacioni and Lincoff, 1981; Moser, 1983).

*Extraction*

The fruiting bodies of the wild mushroom (*Hexagonia glabra*) were washed in tap water and then distilled water to remove impurities. Hence, they were air dried in oven at 50°C for 48 h, chopped into pieces, and grinded into powder using mixer grinder. Briefly, 15 g of dried mushroom powder was dipped in 150 mL of 90% ethanol in glass bottles with tightly fitted cap, under shaking condition at room temperature for 3 days. The same procedure was followed for ethyl acetate extract (EAE). In case of water extract (WE), mushroom power was dipped in boiling water for 30 min. All extracts were filtered through Whatman No. 4 followed by Whatman No.1 filter papers, and then filtrates were collected. The solvents from each filtrate of extracts were removed using a rotary vacuum evaporator at 40°C, and hence the extract was lyophilized to dry powder. The powder of extracts (EE, EAE, and WE) was weighed and kept in airtight condition in refrigerator at 4°C for further use. Extracts used for in vitro assays were dissolved in plain RPMI 1640 medium and passed through a 0.22 μm Millipore filter for sterilization. The prepared extract was further diluted with plain RPMI 1640 medium in certain concentrations just prior to use.

*Cell culture*

Three human cervical cancer cell lines, namely HeLa, SiHa, and CaSki, were purchased from NCCS, Pune, India. They were cultured separately in Dulbecco’s Modified Eagle media (DMEM) supplemented with L-Glutamine (Company), 10% v/v fetal bovine serum (company), 100 μg/mL streptomycin (Invitrogen), and 250 IU/mL penicillin (Invitrogen) in 75 square mm tissue culture flasks at 37°C in a humidified atmosphere of 5% CO_2_ to the 80-90% confluence (Freshney, 2015).

*Morphological examination of cancer cells after treatment with EE, EAE, and WE* *Cell morphology study by phase contrast/ bright field microscopy*

Each cancer cell line was grown in 60-mm tissue culture dish and treated with each extract separately at 150 μg/mL of extract concentration for 24 h. Cells were examined under phase contrast /bright field microscopy, and photographs were taken using a Magna-Fire digital camera for analysis. 

*Nuclear morphology by DAPI staining under inverted fluorescent microscope*

DAPI (4’, 6-Diamidino-2-phenylindole) staining was done to observe the treated (150 μg/mL of extract power) cells’ nuclear morphology. The HeLa, SiHa, and CaSki were washed with cold PBS and fixed with 3.7% (w/v) para-formaldehyde in PBS for 10 min at room temperature. After permeabilization, the cells were stained with a DAPI (10 μg/mL) solution at 37^o^C for 30 min. The cells were washed with PBS and were examined under an inverted fluorescent microscope (Olympus, Tokyo, Japan), and photographs were taken using a Magna-Fire digital camera (Optotronics, Goleta, CA, USA) for analysis. 

*Evaluation of apoptosis by DAPI staining under inverted fluorescent microscope*

Apoptosis was evaluated by morphological changes in the nuclear structure of all three treated (150 μg/mL) cell lines stained with DAPI comparing with control set. The apoptotic cells were analyzed and counted under an inverted fluorescent microscope in each experiment, and at least five optical fields were counted in each of them containing a total of 200 cells.

The percentage of apoptotic cells was calculated by the following formula: 

Percentage of apoptotic cells = [number of apoptotic cells / total cells counted (200 usually)] × 100%. 

*Cell proliferation or cytotoxicity assay*

The effects of each mushroom extract on cell proliferation of HeLa, SiHa, and CaSki cell lines were evaluated by using Dimethyl thiazolyl tetrazolium bromide (MTT) assay (Sigma, USA) and in accordance with Mosmann method (Mosmann, 1983) and by implementing some modifications. Briefly, 10×10^3^ cells per well of 96-well culture plate were seeded with fresh DMEM medium, containing 10% FBS and antibiotics, overnight to reach 80% confluency. Then, the culture was washed with 10% PBS, treated at different concentrations (0, 50, 100, 150, 200, and 250 µg/mL of each extract dissolved in DMEM), and incubated at 37°C in 5% of CO_2_ and 95% of air. After 24, 48, and 72 h treatment, cells were washed with PBS, 100 µL of 0.5% MTT solution (dissolved in RPMI 1640) was added to each well, and cultures were further incubated for 3 h. After discarding the media, 100 µL of DMSO was added for dissolving the crystals. The plate was read by using micro plate reader (Bio-Rad) at 570 nm absorbance. For the normal human lymphocytes, which are in suspension, the cytotoxicity was evaluated using the water-soluble MTS (Vorauer et al., 1996) dye.

Growth inhibition rate was determined by the following formula:

Growth inhibition = [ 1- A 570 nm of treated cells / A 570 nm of control cells] × 100%

The concentration which led to a 50% killing (IC_50_) was calculated by plotting a dose response graph of the cytotoxicity values obtained using the formula given below: 

% Cell cytotoxicity = 100 – [(A control - A test / A control) × 100]

Data points represent the mean ± SD in one experiment repeated at least thrice.

*Western blot analysis for study of gene expression*

Each cancer cell line (2×10^5^) was treated with 150 µg/mL of each extract separately for 24 h. After treatment, cells were lysed with RIPA buffer (Abcam). The effect of treatment on the expression of certain genes as proteins, such as p53, and on apoptotic proteins, such as Bcl-2, caspase-3, and caspase-9 (Santacruze Biotechnology, USA), were measured. Proteins were detected by incubation with the corresponding primary antibodies, and antibodies followed by blotting with the HRP-conjugated secondary antibody. The blots were then detected using Luminol (Bio-Rad), and intensity of bands of each protein was measured by Image J. 

*Cell cycle analysis*

 CaSki cells (7.5x10^5^) were seeded in 100 mm dishes, and cultured in DMEM containing 10% FBS for 24 h. Then, cells were incubated with EE (50 and 150 μg/ml) and positive control Cisplastin (50 μg/ml) separately at 37°C and 5% CO_2_ for 24 h, where DMEM used as vehicle control. After incubation, the cells were harvested, washed with Dulbecco’s PBS containing 1% FBS, and re-suspended in 50 μg/ml propidium iodide (PI). Samples were analyzed on a fluorescence-activated cell sorting (FACS Calibur BD Bioscience, USA).The fractions of cells in the various phases of the cell cycle (G0/G1, S, and G2/M) were shown as a percentage of the total cells analyzed.

## Results

*Yield of extracts*

**Figure 1:**
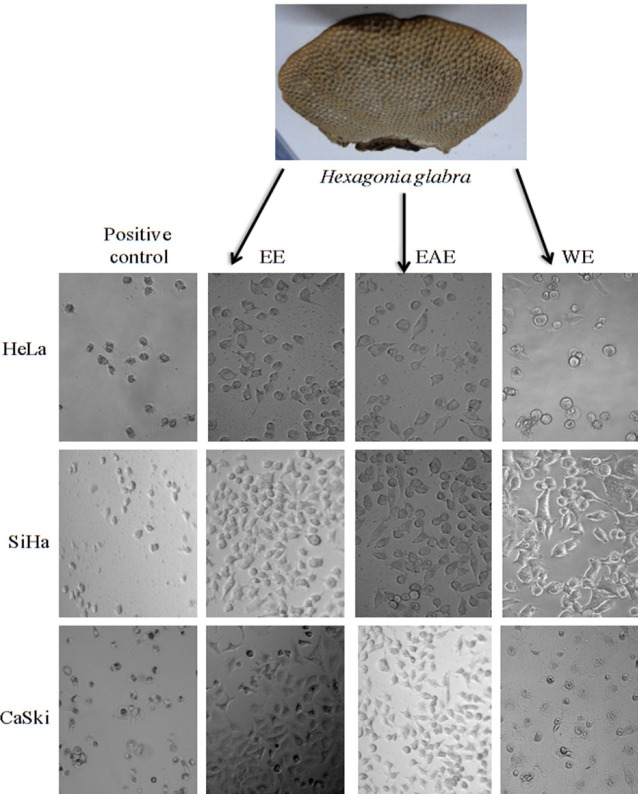
Morphological Changes of Human Cancer Cell Lines Treated with EE, EAE, WE (150μg/mL) and Negative Control under Phase Contrast Microscope at 24 h. HeLa, SiHa and CaSki cells have no changes in Negative Control; but treated cells are round, degenerated and dead

The yields of EE, EAE, and WE of *P. glabra *(Figure 1) were 7.79, 2.21, and 5.46% (W/W), respectively. 

*Cell morphological study of all three cell lines under phase contrast microscopy*

Figure 1 showes that HeLa cells treated with 150 µg/mL of EE resulted in more round, shrunken, and membrane blabbing. The dead cells and cell debris were also observed. The cells treated with 150 µg/mL of EAE and WE, separately, were similar to fate of HeLa cells. Other two cell lines (SiHa and CaSki), when exposed to earlier concentration of these extracts at 24 h, showed the same trends. All three types of cancer cells treated with Adriamycin (positive control) became round shape, while many cells were dead (Figure 1). 

*Nuclear morphological study of all three cell lines under florescence microscopy*

**Figure 2:**
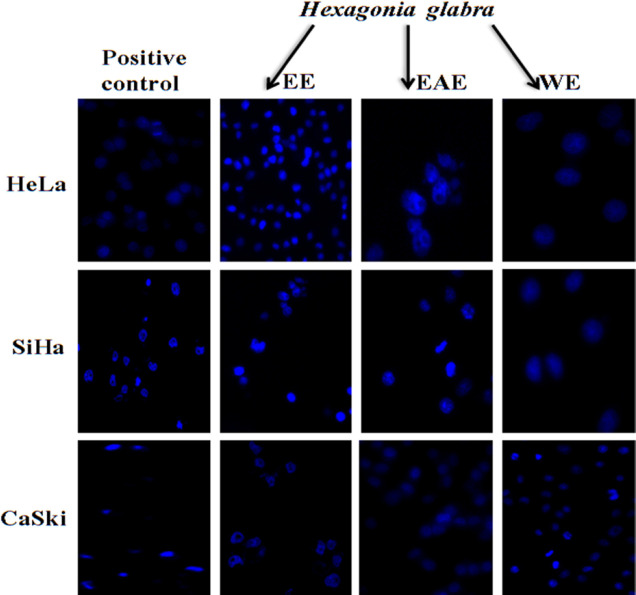
Nuclear Morphology of Human Cancer Cell Lines Treated with EE, EAE, WE (150 μg/mL), Negative Control under Inverted Fluorescence Microscope at 24 h. HeLa, SiHa and CaSki nuclei are condensed, highly fluoresced and lobed under exposure of mushroom extracts and Negative Control exhibited no nuclear changes

Nuclei of the control/ untreated HeLa, SiHa, and CaSki1cells appeared normal taking light blue color and were round and homogeneous, while nuclei which were treated with EE, EAE, and WE, separately, were condensed and in few cases were irregular and fragmented (Figure 2). It was revealed that all four extracts were effective on inducing apoptosis of all three cell lines. 

*Evaluation of apoptosis by DAPI staining under inverted fluorescent microscope*

**Figure 3:**
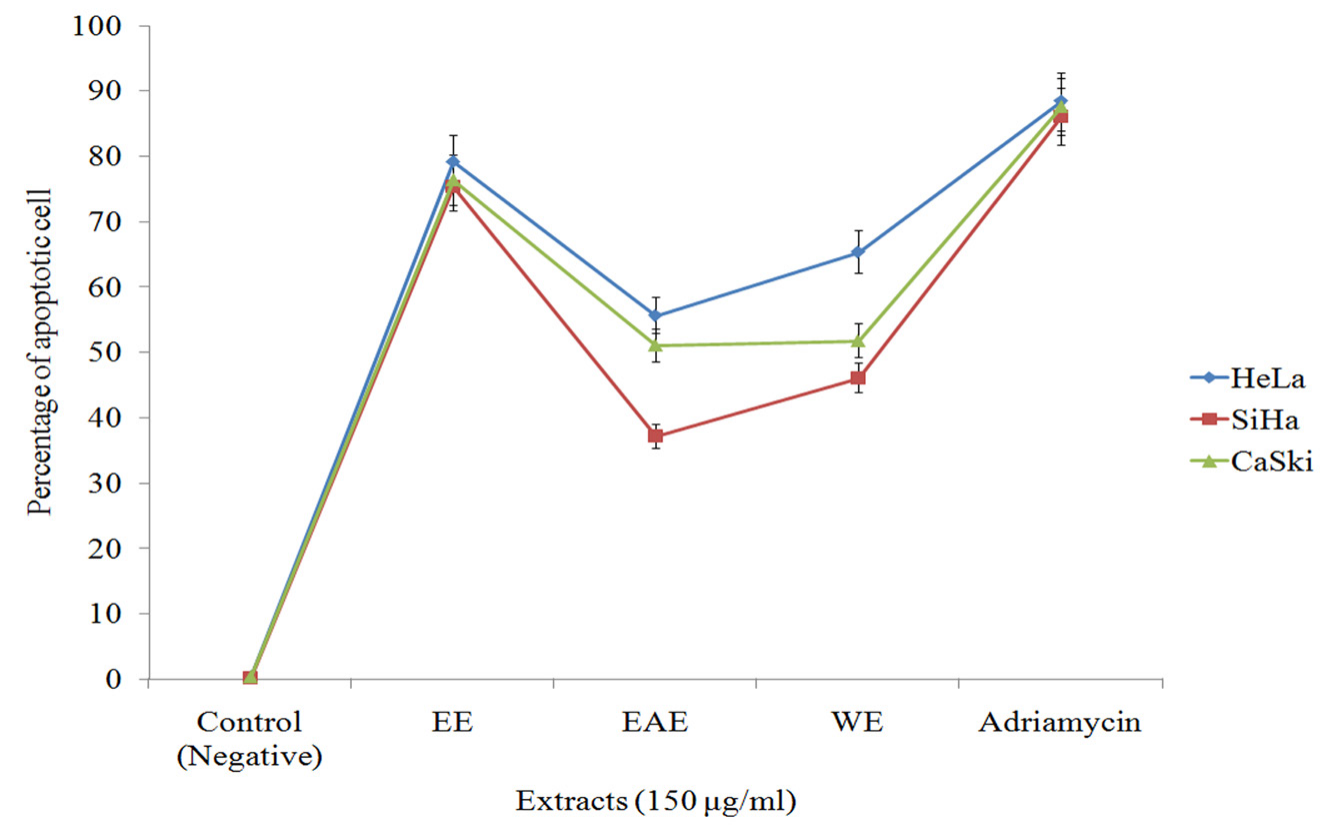
Effect of Mushroom Extracts (EE, EAE and WE) on the Rate of Apoptosis of Cervical Cancer Cell Lines (HeLa, SiHa and CaSki) Compared with Positive and Negative Control. X-axis denotes different mushroom extracts (150 μg/mL concentration) and Y-axis denotes percentage of apoptotic cells. Standard error (SE±) bar inserted (p<0.05)

The percentages of apoptotic cells of HeLa, SiHa, and CaSki were 79.23, 75.42, and 76.36 %, respectively, at 150 μg/mL concentrations of EE. At positive control (Adriamycin), the percentages were 88.34, 86.09, and 87.51%, respectively (Figure 3). The percentages of apoptotic cells of HeLa, SiHa and CaSki at 150 μg/mL concentrations of EAE were 55.67, 37.12, and 51.07 %, respectively. The percentages were 65.32, 46.12, and 51.78%, respectively, at 150 μg/mL concentrations of WE. It indicated that exposure of all three cell lines to these mushroom extracts (EE, EAE and WE) for 24 h resulted in apoptosis (Figure 3). 

*Anti proliferative/cytotoxicity effect of mushroom extracts against HeLa, SiHa, and CaSki cell lines based on MMT assay*

We investigated the effect of each of three extracts on cell proliferation of three cell lines. According to Table 1, the proliferation of all cell types was reduced gradually as dosage of EE increased gradually from 50 to 250 µg/mL. The percentages of cell growth inhibition in HeLa, SiHa, and CaSki cells treated with EE at 24 h at 50 µg/mL (the lowest dosage) were 45.79±4.11, 41.66±4.03, and 36.72±2.67, respectively. At 150 µg/mL (the moderate dosage), the percentages were 74.23±7.45, 62.31±5.97, and 54.23±5.04. At 250 µg/mL (the highest dose), the percentages were 94.25±8.11, 90.02±8.67, and 85.43±6.21, respectively. The positive control (Adriamycin 5 µg/mL) inhibited all three cell lines by 99.01±10.45, 98.64±9.11, and 95.34±9.79%, respectively. Data from Table 2 showed that the proliferation of all cell types were reduced gradually as dosage of EAE increased (50-250 µg/mL). The percentages of cell growth inhibition in HeLa, SiHa, and CaSki cells treated with EAE at 24 h at 50 µg/mL (the lowest dosage) were 15.67±2.11, 10.15±1.86, and 9.12±1.06, while at 150 µg/mL (the moderate dosage), the percentages were 41.56±3.79, 34.16±3.12, and 27.78±2.67, respectively. Regarding 250 µg/mL dosage (the highest dosage), the percentages of cell growth inhibition in studies cells were 56.01±4.92, 49.56±5.23, and 45.11±4.21, respectively (Table 2). As it is clear from Table 3, the percentages of cell growth inhibition in HeLa, SiHa, and CaSki cells treated with WE at 24 h at 50 µg/mL (the lowest dosage) were 21.81±2.34, 18.43±1.75, and 25.88±2.59, while at 150 µg/mL (the moderate dosage) were 38.56±3.78, 34.19±3.90, and 49.23±4.15. At 250 µg/mL dosage (the highest dosage), the percentages were 67.90±6.01, 51.23±5.56, and 75.56±6.45, respectively (Table 3). It was noted that HeLa was more sensitive to EE and EAE, while CaSki cell line was more sensitive to WE than other two cell lines (Tables 1-3). The percentages of cell growth inhibition in all treated (150 μg/mL of EE, EAE, WE) cell lines were increased gradually when incubation time was increased from 24 to72 h (Data not shown) . IC_50_ values of EE against HeLa, SiHa, and CaSki were 60.45±6.21, 99.89±7.45, and 140.32±15.32 µg/mL, respectively (Table 1). IC_50_ values of of EAE against these cell lines were 204.14±17.45, 254.56±20.11, and 262.01±22.78, respectively (Table 2). IC50 values of of WE against these cell lines were 220.34±17.23, 248.21±19.67, and 152.66±11.44, respectively (Table 3). All these extracts at the highest dosage (500 µg /mL) showed no cytotoxicity against the normal human lymphocytes up to 72 h (Data not shown).

*Study of induction of apoptosis using gene expression in Western blotting assay*

**Figure 4.**
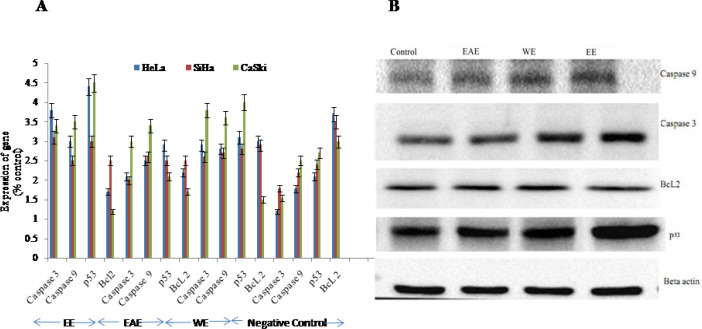
Expression of Genes Analyzed by Western Blotting Assay of the HeLa. SiHa nad CaSki cells treated by EE, EAE, WE and compared with Negative Control. A. The intensity of protein bands of expressed genes measured by Image J software. Standard error (SE±) bar inserted (p<0.05), B. Bands of proteins of CaSki cells in Western blotting after treatment with EE, EAE and WE (150 μg/mL in each)

We found that treatment with EE, EAE, and WE at the concentration of 150µg/mL decreased *BcL2 *gene expression (Figure 4), while increased the expression of apoptosis genes, namely caspase 3 and caspase 9 in all three cell lines in vitro. Moreover, gene *p53* of cell lines was up-regulated by EE, EAE, and WE (Figure 3). Comparative analysis among these three extracts for their ability to upregulate *caspase 3, 9* and *p53* genes and down regulate *BcL2 *genes in all three cancer cell lines, it was reflected that EE extract was best followed by WE and EAE respectively (Figure 4). 

*Cell cycle arrest by EE extract*

**Figure 5:**
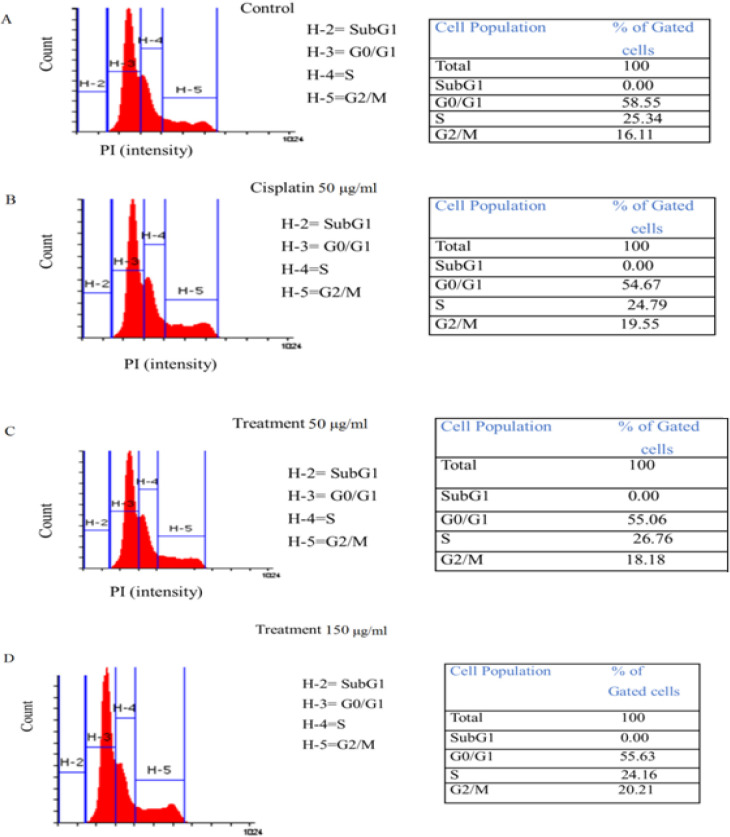
Cell Cycle Analysis of CaSki by Flow Cytometry A. Vehicle (Negative) control; B. Positive control (Cisplastin 50 μg/mL); C. Treatment (EE, 50 μg/mL); D. Treatment (EE, 150 μg/mL).

The cell cycle distribution of CaSki cells was subsequently investigated using flow cytometry with the PI staining method. Based on Figure 5, the percentage of cells distributed in the G2/M phase of the cycle was 16.11% for the negative control (DMEM vehicle) (Figure 5A), and it was 19.55% in case of positive control (cisplastin 50 μg/ml). The percentage of cells distribution in G0/G1 was 54.67% (Figure 5B). Upon treatment with EE at concentrations of 50 and 150 μg/ml, the percentages of cells distributed in the G2/M phase increased gradually to 18.18% and 20.21% (Figure 5C, 5D), respectively. Treatment of the cells with EE at 50 and 150 μg/ml concentrations also led to a gradual decrease in the cell distribution at the G0/G1 and S phases compared with the negative control.

**Table 1:** Percentage of Cell Viability and Inhibition of HeLa, SiHa and CaSki Cell Lines by EE at 24h Calculated IC_50_ Value

Concentration of EE (µg/mL)	Cell viability ± SD (%)			Inhibition± SD (%)			IC_50_± SD value of EE		
	HeLa	SiHa	CaSki	HeLa	SiHa	CaSki	HeLa	SiHa	CaSki
50	54.21±5.12b	58.34±6.60b	63.28±5.23b	45.79±4.11e	41.66±4.03e	36.72±2.67e	60.45±6.21	99.89±7.45	140.3±15.32
100	42.08±3.44c	49.84±4.96c	64.55±7.08b	57.92±5.12d	50.16±4.97d	44.97±4.78d			
150	25.77±3.11d	37.69±2.90d	45.77±4.44c	74.23±7.45c	62.31±5.97cd	54.23±5.04cd			
200	14.37±1.95e	28.77±2.89e	36.57±4.58d	85.63±8.21b	71.23±6.23c	63.43±5.1c			
250	05.75±0.97f	09.98±2.11f	14.57±3.12e	94.25±8.11a	90.02±8.67b	85.43±6.21b			
Control (negative)	100a	100a	100a	0	0	0			
Positive control (Adriamycin 5 µg/mL)	0.99±0.15g	0.36±0.05g	4.66±0.99f	99.01±10.45a	98.64±9.11a	95.34±9.79a			

Note: Each data is Mean± SD (p<0.05) of three independent experiments. Same letter in the same row indicates statistically no different but different letter indicates they are statistically different as per Duncan analysis (p=0.05 level)

**Table 2:** Percentage of Cell Viability and Inhibition of HeLa, SiHa and CaSki Cell Lines by EAE at 24h Calculated IC_50_ value

Concentration of EAE (µg/mL)	Cell viability ± SD (%)			Inhibition± SD (%)			IC_50_± SD value of EAE		
	HeLa	SiHa	CaSki	HeLa	SiHa	CaSki	HeLa	SiHa	CaSki
50	84.33±7.67b	89.85±8.08b	90.88±9.56b	15.67±2.11f	10.15±1.86e	9.12±1.06f	204.14±17.45	254.56±20.11	262.01±22.78
100	76.77±6,99c	81.33±7.78c	84.88±7.90bc	23.23±2.98e	18.67±1.90d	15.12±2.52e			
150	58.44±5.45d	65.84±5.23d	72.22±6.98c	41.56±3.79d	34.16±3.12c	27.78±2.67d			
200	50.45±5.21d	55.12±4.10e	65.55± 6.55d	49.54±4.11c	44.78±4.16b	34.45±3.56c			
250	43.99±3.12e	50.44±4.34e	54.89±5.63e	56.01±4.92b	49.56±5.23b	45.11±4.21b			
Control (negative)	100a	100a	100a	0	0	0			
Positive control (Adriamycin 5 µg/mL)	1.91±0.41f	2.44±0.52f	2.92±0.71f	98.19±11.45a	97.56±10.12a	97.08±11.79a			

Note: Each data is Mean± SD (p<0.05) of three independent experiments. Same letter in the same row indicates statistically no different but different letter indicates they are statistically different as per Duncan analysis (p=0.05 level)

**Table 3:** Percentage of Cell Viability and Inhibition of HeLa, SiHa and CaSki Cell Lines by WE at 24h Calculated IC_50_ Value

Concentration of WE (µg/mL)	Cell viability ± SD (%)			Inhibition ± SD (%)			IC_50_ ± SD value of WE		
	HeLa	SiHa	CaSki	HeLa	SiHa	CaSki	HeLa	SiHa	CaSki
50	78.19±6.75b	81.57±7.67b	74.12±6.89b	21.81±2.34f	18.43±1.75e	25.88±2.59f	220.34±17.23	248.21±19.67	152.66±11.44
100	68.67±6.12c	73.88±6.99b	62.24±5.90c	31.33±3.11e	26.12±2.34d	37.76±3.65e			
150	61.44±6.11c	56.10±5.58c	50.77±4.98d	38.56±3.78d	34.19±3.90c	49.23±4.15d			
200	53.11±4.22d	61.00±6.01c	42.24±4.78e	46.89±4.89c	39.00±4.02c	57.76±4.90c			
250	32.10±3.87e	48.77±4.05d	24.44±2.35e	67.90±6.01b	51.23±5.56b	75.56±6.45b			
Control (negative)	100a	100a	100a	0	0	0			
Positive control (Adriamycin 5µg/mL)	1.09±0.55f	1.89±0.65e	0.11±0.66f	98.91±9.22a	98.11 ±9.17a	99.89 ±10.12a			

Note: Each data is Mean± SD (p<0.05) of three independent experiments. Same letter in the same row indicates statistically no different but different letter indicates they are statistically different as per Duncan analysis (p=0.05 level)

## Discussion

Earlier occurrence and distribution of Hexagonia speciosa were recorded in China (Zhao, 1998), but there remains a lacuna in proper record of distribution of H. glabra in China and India as well. Its collection from fallen logs in 24-Parganas district of West Bengal, India was tentatively first place where it was collected and yield of EE, EAE and WE of Hexagonia glabra in our experiment were taken but there are no reports of such yields earlier, yet. The isolation and structure determination of a series of oxygenated cyclohexanoids were performed by Jiang et al., (2009) from Hexagonia speciosa. They include speciosins, 5H-furan-2-one me-tabolite, 5′-O-acetylaporpinone A, and aporpinone A. Out of these compounds, speciosin B exhibited cytotoxicity against some cancer cell lines. IC_50_ values range from 0.23–3.30 μM (Jiang et al., 2009; Jiang et al., 2011). Silva et al., (2009) first reported antitumor activity of methanolic extract (ME) of the Hexagonia papyraceae against K562 (Human chronic myeloid leukemia cell) and Daudi (human Burkitt’s lymphoma cell). ME inhibited more than 60% of the proliferation of both K562 and Daudi cells. The IC_50_ value against K562 was 39.1μg/mL. Literature review indicated that anticancer effect of solvent extract of H. glabra is not tested against any cancer cell line or in animal model by any workers . However, different solvent extracts of other mushrooms have been applied on several cancer cell lines.* Laetiporus sulphureus*, the saprophytic wild, cultivated polypore mushroom and weak parasite of plant, the ethanol extract of it also exhibited anti-proliferative activity against three carcinoma cell lines HeLa, HCT 116 and MCF-7 (Younis et al., 2019). The effects of ethanol extracts from *G. frondosa*,* G. lucidum*, *Hericium erinaceus*, and *L. edodes* fruiting bodies, spores, and cultured broth on cell proliferation and apoptosis in CH72 cancer cells and C50 normal cells were evaluated (Gu and Belury, 2005). Out of these extracts, ethanol extract from *L. edodes* significantly inhibited CH72 cell proliferation, while none of these extracts had effect on normal C50 cells. Cell cycle analysis exhibited that L. edodes extract induced a transient G1 phase arrest in CH72 cells. *Polyozellus multiplex* inhibited cell proliferation in stomach cancer by increased expression of p53 proteins (Lee and Nishikawa, 2003). Lavi et al., (2006) showed that an aqueous polysaccharide extract from the edible mushroom *Pleurotus ostreatus* induced anti-proliferative and pro-apoptotic effects on HT-29 colon cancer cells. The mushroom extract can be an effective therapy for malignant estrogen-independent breast cancer (Asatiani et al., 2011). Hot-water and ethanol extract of Inonotus obliquus has ability to induce apoptosis in human colon cancer (DLD- 1) cells by the prevention of reactive oxygen species (ROS) - induced tissue damage (Hu et al., 2009). Youn et al., (2009) tested the anti-proliferative effects of water extract of *I. obliquus* extract on B16-F10 cells (murine melanoma). The ethanolic extract of the fruiting body of *P. igniarius* was evaluated as the anti-proliferative and anti-metastatic agent against SK-Hep-1 (human hepatocarcinoma) and RHE (rat heart vascular endothelial). The extract inhibited cell growth of both cell lines in a dose-dependent manner, and the IC_50_ values at 48 h were 72 and 103 μg/mL for SK-Hep-1 cells and RHE cells, respectively (Song et al., 2008). The growth of CaSki (Epidermoid cervical carcinoma) cells when treated with the crude dichloromethane extracts of *G. lucidum* was inhibited (Lai et al., 2010). The dichloromethane extract contains flavonoids, terpenoids, phenolics, and alkaloids with anti-human papillomavirus 16 (HPV 16) E6 oncoprotein activity. The methanol extract and its fractions, viz., methylene chloride, ethyl acetate, and n-butanol of Phellinus linteus exhibited anti-angiogenic effects (Lee et al., 2010). Huang et al., (2011) recorded the anti-cancer effect of P. linteus and interpreted its potential mechanism. They showed increase in number and activity of T cell and NK cell. *A. campestris* extract inhibited the growth of three cell lines, namely HeLa cells, A549 cells (human lung carcinoma), and LS174 cells (human colon carcinoma) (Kosanić et al., 2017).

In this study, we screened three extracts of H. glabra mushroom (EE, EAE, and WE) against three cervical cancer cell lines, namely HeLa, SiHa, and CaSki by MTT assay. We noted antiproliferation and apoptotic activity of three extract against all three cell lines. In the laboratory (Ghosh, 2015), ME, EE, and WE of wild Calocybe indica were tested for anticancer effect on MCF 7 and Ewing Sarcoma cell line, and similarly antiproliferation and apoptotic effect of ME and EE of Agaricus bisporus against CaSki cell line were reported (Ghosh et al., 2018). EAE of *C. indica* was applied against HeLa and CaSki, and it was found that this extract caused changes in cancer cells and nuclei, apoptosis and inhibited proliferation of these two cell lines and this mushroom extract increased the expression apoptotic genes (*caspase 3, 9*), tumor guard gene *p53* and decreased the pro-apoptotic gene *BcL2* (Ghosh et al., 2019). In the present experiment, all mushroom extracts showed morphological changes in all cancer cells and their nuclei, and antiproliferative and apoptotic activity. Increase in concentration of all extracts increased anticancer activity. Antiproliferative effect, including morphological changes, against BXPC3 cell line was also noted (Chen et al., 2009). However, Yu et al., (2012) exhibited that antroquinonol, a ubiquinone derivative isolated from the same mushroom, inhibited cell proliferation of PANC-1 and AsPC-1 cells in dose-dependent manners as we noted in MTT assay. In this study, in three cell lines morphological changes like round and shrunk, and membrane blebbing (bulbing) and the reduction of cell confluence were noted in all extracts. Similar result was found when HeLa cells treated with 500 µg/mL /750 µg/mL of methanolic extract of Agaricus bisporus for 24h at the same conditions (Ghosh et al., 2018). All extracts also modulated the expression of some genes (*Caspase 3, 9, p53,* and *BcL2*). These results were also validated by other researchers investigated other cancer cell lines and other mushrooms (Gu and Belury, 2005; Zaidman et al., 2005). The ethanol and ethyl acetate extracts of Coprinus comatus inhibited LNCaP cells of Prostate cancer (Zaidman et al., 2008). Researchers noted that that these extracts inhibited dihydrotestosterone-induced LNCaP cell viability and arrested cell cycle at G1 phase. In the cell cycle experiments performed in the present study, EE arrested cell cycle at the G2/M checkpoint of CaSki cells. Similarly, Jiang and Sliva (2010) observed that the methanolic extract of myco-complex induced significant cell cycle arrest at the G2/M phase. Furthermore, cell cycle arrest at G2/M was induced by *A. blazei* in gastric epithelial cells (Jin et al., 2006), and by cordycepin isolated from C. sinensis in bladder cancer cells (Lee et al., 2009).It is noteworthy that different extracts from *G. lucidum *demonstrated specific effects on cell cycle progression. Extracts from* G. lucidum *were shown to induce cell cycle arrest at the G0/G1 phase in breast cancer cells (Jiang et al., 2006), where as arrest at the G2/M phase was induced in prostate (Jiang et al., 2004), hepatoma (Lin et al., 2003) and bladder (Lu et al., 2004) cancer. 

The ethyl acetate and culture broth extracts of this also showed antiproliferative activity against MCF7 cells. One study showed that IC50 value was 76 μg/ mL for culture broth extract and 32 μg/mL for ethyl acetate extract (Asatiani et al., 2011). Among mushroom extracts, an ethanol extract may find the most extensive application. In this study, we also found that EE was the best among three extracts as anticancer agent against three cervical cell lines. On the other hand, we detected that HeLa was more sensitive to EE and EAE, while CaSki cell line was more sensitive to WE than other two. Similar to our findings, one researcher found that ethanol extracts of Pleurotus florida and Calocybe indica caused apoptosis in T24 cell line (Selvi et al., 2011). 

In conclusion, all extracts of this mushroom were active in inhibiting the growth of all three cervical cell lines with fair percentage at 24 h. Among three extracts, EE showed maximum anti-proliferation activity against all three cell lines (i.e. HeLa, SiHa, and CaSki). Their activities induced apoptosis and upregulation of apoptotic genes and downregulation of pro-apoptotic genes. In addition, it was detected that EE extract arrested cell cycle at G2/M point. Further studies are suggested to examine the mycochemistry of this mushroom extracts and their application in animal model for next generation anticancer drug development. 
